# Molecular Mechanisms, Diagnoses, and Treatments of Respiratory Diseases

**DOI:** 10.3390/biomedicines13010004

**Published:** 2024-12-24

**Authors:** Te-Chun Shen

**Affiliations:** 1 School of Medicine, China Medical University, Taichung 404, Taiwan; chestshen@gmail.com; Tel.: +886-4-22052121; 2 Department of Internal Medicine, China Medical University Hospital, Taichung 404, Taiwan; 3 Department of Internal Medicine, Chu Shang Show Chwan Hospital, Nantou 557, Taiwan

The Special Issue “Molecular Mechanisms, Diagnoses, and Treatments of Respiratory Diseases” in the journal *Biomedicines* compiles critical advancements in the understanding of respiratory diseases, focusing on their molecular mechanisms, diagnostic approaches, and therapeutic strategies. Inspired by the profound global impact of Coronavirus Disease 2019 (COVID-19), this Special Issue highlights the urgent need for deeper insights into respiratory diseases to facilitate effective clinical interventions and improve patient outcomes. This collection includes original research articles and comprehensive reviews exploring diverse aspects of respiratory diseases [1,2,3,4,5,6,7,8]. Collectively, these studies contribute to a more holistic understanding of respiratory pathogenesis and pave the way for the development of innovative strategies for early diagnosis and precision treatments. Notably, there has been a remarkable surge in the publication of research over the past one to two years addressing a wide range of respiratory diseases, including COVID-19 [9,10,11,12,13,14], asthma and chronic obstructive pulmonary disease (COPD) [15,16,17,18], interstitial lung disease (ILD) [19,20,21], lung cancer [22,23,24,25,26,27,28,29,30], bronchiectasis [31,32,33], pulmonary infection [34], pulmonary hypertension [35,36], and other respiratory disorders [37,38,39,40].

Within the context of COVID-19, several studies in this Special Issue focus on pulmonary fibrosis and other long-term sequelae following SARS-CoV-2 infection. For instance, “Long-Term Radiological Pulmonary Changes in Mechanically Ventilated Patients with Respiratory Failure due to SARS-CoV-2 Infection” examines the radiologic and biochemical changes in intensive care unit (ICU) patients with COVID-19 who received mechanical ventilation [1]. This prospective study from Romania highlights the association of specific biomarkers with pulmonary outcomes, including fibrosis, thus suggesting the need for ongoing radiological monitoring in patients with severe COVID-19. Similarly, “Clinicopathological Outlines of Post-COVID-19 Pulmonary Fibrosis Compared with Idiopathic Pulmonary Fibrosis” offers a comprehensive review of the distinguishing clinical, radiologic, and histological features between post-COVID pulmonary fibrosis and idiopathic pulmonary fibrosis (IPF) [2]. This comparison will aid clinicians and researchers in better identifying patient subgroups suited for anti-fibrotic therapies, ultimately guiding future therapeutic approaches.

Furthermore, this Special Issue includes critical analyses of oxygen therapy in the management of hypoxemic respiratory failure. The article “Monitoring the Efficacy of High-Flow Nasal Cannula Oxygen Therapy in Patients with Acute Hypoxemic Respiratory Failure in the General Respiratory Ward: A Prospective Observational Study” investigates the effectiveness of high-flow nasal cannula (HFNC) oxygen therapy [3]. This study supports the use of HFNC as a viable rescue therapy and introduces electrical impedance tomography as a promising tool for monitoring ventilation distribution in real time. These insights could refine respiratory failure management protocols, particularly in general wards where access to advanced ICU resources may be limited.

In COPD research, the articles cover innovative therapeutic targets and risk assessment strategies. For example, “KCa2 and KCa3.1 Channels in the Airways: A New Therapeutic Target” identifies specific potassium channels in the airways as potential therapeutic targets for muco-obstructive diseases [4]. This study explores the roles of KCa2 and KCa3.1 channels in lung physiology and pathology, indicating that modulators of these channels could provide new treatment options for COPD. Additionally, “Associated Factors of Pneumonia in Individuals with Chronic Obstructive Pulmonary Disease Apart from the Use of Inhaled Corticosteroids” delves into pneumonia risks among COPD patients, extending beyond the known association with inhaled corticosteroids (ICSs) [5]. This research suggests that other factors in COPD patients can contribute to pneumonia risk, thereby guiding more nuanced ICS-based therapeutic approaches.

The section on ILD covers the complex interplay between autoimmune diseases and respiratory conditions. In “Better Safe than Sorry: Rheumatoid Arthritis, Interstitial Lung Disease, and Medication—A Narrative Review”, the review examines the dual risks of ILD associated with both rheumatoid arthritis (RA) and its treatments [6]. The article highlights the potential for RA therapies, such as disease-modifying antirheumatic drugs, to induce or exacerbate ILD. This study emphasizes the need for cautious and personalized treatment regimens for RA patients with pulmonary complications, which could help clinicians prevent adverse respiratory outcomes. Another relevant article, “Risk of Acute Myocardial Infarction in Pneumoconiosis: Results from a Retrospective Cohort Study”, underscores the connection between pneumoconiosis and cardiovascular risks [7]. This population-based study from Taiwan identifies a higher incidence of acute myocardial infarction among pneumoconiosis patients, advocating for integrated cardiovascular monitoring and preventive strategies in this patient group.

This Special Issue also addresses pediatric respiratory health, focusing on obstructive sleep apnea (OSA) in children. “Diagnosis and Treatment of Sleep Apnea in Children: A Future Perspective Is Needed” highlights the current challenges and limitations in diagnosing and treating pediatric OSA [8]. Despite significant repercussions on children’s cardiovascular, metabolic, and neurological health, pediatric OSA remains underdiagnosed and undertreated. This article calls for a re-evaluation of diagnostic criteria, including an updated definition of disease severity and the exploration of potential biomarkers for risk assessment. The authors advocate for a shift towards personalized medicine in the management of pediatric OSA, which would enhance treatment efficacy and address the heterogeneity of disease presentation in this population.

In conclusion, this Special Issue provides a multifaceted exploration of respiratory disease research, from the cellular and molecular underpinnings to clinical applications in diagnosis and treatment. Collectively, these studies highlight emerging therapeutic targets, innovative diagnostic techniques, and comprehensive disease management approaches. Looking forward, advancements in molecular and translational respiratory research will likely continue to drive forward personalized medicine, improving outcomes for patients with diverse respiratory conditions. This Special Issue aims to serve as a valuable resource for healthcare providers, researchers, and policymakers as they work to reduce the burden of respiratory diseases on a global scale. By advancing knowledge and promoting collaboration across disciplines, we hope to contribute to a future where respiratory health is proactively managed, enabling better quality of life and survival outcomes for affected patients.
